# *Swimming in Deep Water*: Zebrafish Modeling of Complicated Forms of Hereditary Spastic Paraplegia and Spastic Ataxia

**DOI:** 10.3389/fnins.2019.01311

**Published:** 2019-12-10

**Authors:** Valentina Naef, Serena Mero, Gianluca Fichi, Angelica D'Amore, Asahi Ogi, Federica Gemignani, Filippo M. Santorelli, Maria Marchese

**Affiliations:** ^1^Neurobiology and Molecular Medicine, IRCCS Stella Maris, Pisa, Italy; ^2^Department of Biology, University of Pisa, Pisa, Italy; ^3^Struttura Complessa Toscana Sud (Sede Grosseto), Istituto Zooprofilattico Sperimentale del Lazio e Toscana M. Aleandri, Grosseto, Italy; ^4^Department of Neurology, The F.M. Kirby Neurobiology Center, Boston Children's Hospital, Harvard Medical School, Boston, MA, United States; ^5^Department of Veterinary Sciences, University of Pisa, Pisa, Italy

**Keywords:** zebrafish, hereditary ataxia (HA), hereditary spastic paraplegia (HSP), neurodegenerative disorders, motor neuron disease

## Abstract

Hereditary spastic paraplegia (HSP) and hereditary ataxia (HA) are two groups of disorders characterized, respectively, by progressive dysfunction or degeneration of the pyramidal tracts (HSP) and of the Purkinje cells and spinocerebellar tracts (HA). Although HSP and HA are generally shown to have distinct clinical-genetic profiles, in several cases the clinical presentation, the causative genes, and the cellular pathways and mechanisms involved overlap between the two forms. Genetic analyses in humans in combination with *in vitro* and *in vivo* studies using model systems have greatly expanded our knowledge of spinocerebellar degenerative disorders. In this review, we focus on the zebrafish (*Danio rerio*), a vertebrate model widely used in biomedical research since its overall nervous system organization is similar to that of humans. A critical analysis of the literature suggests that zebrafish could serve as a powerful experimental tool for molecular and genetic dissection of both HA and HSP. The zebrafish, found to be very useful for demonstrating the causal relationship between defect and mutation, also offers a useful platform to exploit for the development of therapies.

## Introduction

The term hereditary spastic paraplegia (HSP) refers to a large group of clinically and genetically heterogeneous neurodegenerative diseases, essentially characterized by progressive spasticity and weakness, mainly affecting the lower limbs (Bellofatto et al., 2019; Boutry et al., 2019a; Peng et al., 2019). The clinical classification of HSP includes pure and complicated forms (Harding, 1983), the latter defined by the presence of additional extra-neurological and neurological symptoms, including minor sensory disturbances and hypertonic bladder, retinal changes, and gastroesophageal reflux with persistent vomiting. At times, however, the clinical manifestations can be relatively homogenous within single families (Hensiek et al., 2015; Boutry et al., 2019a; Elert-Dobkowska et al., 2019; Peng et al., 2019). Genetically, HSP can be inherited by any mode of inheritance: autosomal-dominant (AD-HSP), autosomal-recessive (AR-HSP), X-linked, and maternally inherited forms have been described, the latter mainly presenting with complicated clinical phenotypes (Parodi et al., 2017). The prevalence of HSP has been estimated to range from 1.2 to 9.6 per 100,000 individuals (Ruano et al., 2014). Due to the vast genetic diversity of HSP, researchers have turned to the zebrafish, considered a potentially powerful experimental tool for uncovering genotype/phenotype correlations, on account of its having a nervous system organization very similar to that of humans. Pure HSP is characterized by slowly progressive lower extremity spasticity and weakness often associated with urinary disturbances and sensory abnormalities, and it usually shows autosomal dominant inheritance.

Pathologically, the neurodegeneration characterizing HSP is a progressive distal axonopathy of the corticospinal tract and posterior columns in the spinal cord (Salinas et al., 2008; Parodi et al., 2017; Synofzik and Schüle, 2017), in other words a dying-back degeneration that progresses from the distal end of the axons (Boutry et al., 2019b). The axons involved in this degeneration are commonly characterized by a unique, highly polarized architecture (Boutry et al., 2019b). This evidence, together with the discovery that genes involved in axonal transport, intracellular trafficking, and mitochondrial functions are implicated in HSP, suggests that abnormalities in these processes play a role in HSP. However, in complicated HSP, the fact that the neurodegeneration can also involve the cerebellum, cerebral cortex, corpus callosum, and basal ganglia (Hensiek et al., 2015) suggests the involvement of other pathological mechanisms (Boutry et al., 2019b). The identification of other HSP causative genes, together with the discovery of the function of the related proteins, has made it possible to hypothesize at least 10 functional “modes of action” that could play a role in HSP pathogenesis, and also appear to be involved in other neurological disorders: namely, dysfunction of axonal transport, abnormal membrane trafficking and organelle shaping, abnormal endosome membrane trafficking and vesicle formation, oxidative stress, abnormal lipid metabolism, abnormal DNA repair, dysregulation of myelination, autophagy, impairment of axonal development, and abnormal cellular signaling in protein morphogenesis (Lo Giudice et al., 2014; Boutry et al., 2019b). The various forms of HSP, as well as the groups of similar neurodegenerative diseases, such as hereditary ataxia (HA), spinocerebellar ataxia (SCA), autosomal-recessive spinocerebellar ataxia (SCAR), and spastic paraplegia, can be due to mutations in either the spastic paraplegia gene (SPG) or the spastic ataxia genes (SPAX). Clinically, they can present as pure or complicated phenotypes (Synofzik and Schüle, 2017). Formally, HSP and HA are characterized, respectively, by progressive dysfunction or degeneration of the pyramidal tracts (HSP) and of the Purkinje cells and spinocerebellar tracts (HA) (Synofzik and Schüle, 2017). In recent years, genes that cause both cerebellar and pyramidal phenotypes have been discovered, and some genes classified among the HA causative genes have been found to cause HSP phenotypes, too, and vice versa (Galatolo et al., 2018). Furthermore, it is possible that HSP and HA could also share certain pathological mechanisms and cellular pathways. For all these reasons, a new classification of ataxia-spasticity spectrum (ASS) genes has recently been proposed (Synofzik and Schüle, 2017). In the next generation-sequencing era, more than 80 known SPG causative genes and 85 different spastic gait disease loci have been identified as responsible for HSP, some of them very rare and found in few families. However, unusual phenotypes have been described and a larger than expected continuum between the different forms of HSP and other neurodegenerative diseases has been demonstrated (Novarino et al., 2014; Hensiek et al., 2015; Klebe et al., 2015; D'Amore et al., 2018; Galatolo et al., 2018).

In recent years, the zebrafish has emerged as an attractive model for studying human genetic disorders such as HSP, in spite of the existence of key differences between neuroanatomy of the human vs. the zebrafish motor system (Babin et al., 2014), for example, the absence of corticospinal and rubrospinal tracts in the zebrafish central nervous system (CNS). Although this model is limited by its capacity to reproduce only partially the spasticity and the key diagnostic clinical features of the human HSP disorders, the zebrafish remains an indispensable vertebrate model and a valuable tool for the molecular and genetic dissection of HSP mechanisms *in vivo* (Patten et al., 2014). Indeed, the zebrafish offers several key embryological and experimental advantages, including its small size and the optical transparency that allows visualization of cell- and system-level processes in early developmental stages; it also offers a range of quantifiable behavioral responses, which facilitates functional studies. However, the real power of the zebrafish lies in the availability of a very rich collection of mutants and transgenic lines in which is possible to observe the cellular and subcellular events leading to the pathology (Orger and de Polavieja, 2017; Basnet et al., 2019). In this way, the functional connection between a human pathological mutation and disease can be tested experimentally, provided an ortholog with a conserved function has been identified in the model organism (Dahlem et al., 2012; Hwang et al., 2013).

**Figure 1:**
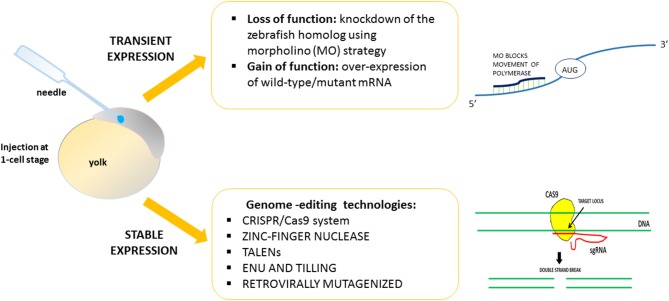
Strategies to validate *in vivo* the function of genes related to HSPs.

Using zebrafish, it is easy to induce gain or loss of function of the HSP-related genes. By exploiting strategies designed to obtain transient or stable genetic changes, it is possible to characterize and define *in vivo* the function and the role of genes found to be mutated in HSP patients (Figure 1). The majority of previous works reported a transient knockdown approach involving the use of antisense oligonucleotides (morpholinos, MOs) (e.g., Nasevicius and Ekker, 2000), in order to validate the effects of gene variants through morphant phenotype rescue experiments based on injection of the human, or zebrafish, wild-type transcript and the mutated version. On the contrary, few mutant genetic models of HSP have been generated. Generally, the lack of function of HSP-related genes leads to major motor neuron defects with greatly impaired locomotion. The morphant phenotype can be rescued through injection of the human, mouse, or zebrafish wild-type transcript. Use of the zebrafish model would seem to be essential in order to close the gap between *in vitro* studies and more costly (in terms of both money and animal distress) assays in mammals, and also in order to develop possible new therapeutic avenues.

With reference, mainly, to the mode of inheritance-based genetic classification of HSP, we here review the HSP genes studied in zebrafish, recalling the original contributions and highlighting the recent discoveries that might help to elucidate the functional effects of human HSP-related mutations. In addition, to further expand our review, we also consider genes classified as part of the ASS spectrum and not routinely included in the formal HSP classifications.

## Materials and Methods

**Figure 2:**
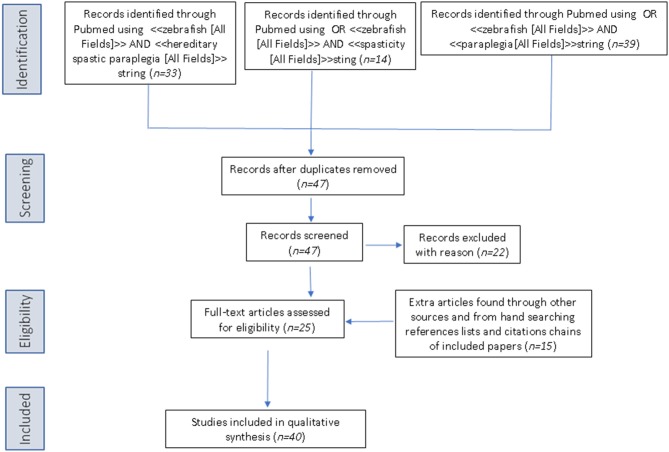
PRISMA flow diagram for literature research.

The PubMed database was queried using the following three search strings: <<zebrafish [All Fields]>> AND <<hereditary spastic paraplegia [All Fields]>>, OR <<zebrafish [All Fields]>> AND <<spasticity [All Fields]>>, OR <<zebrafish>> AND <<paraplegia [All Fields]>>. Articles retrieved by the literature search had to be full-text articles written in English, and they had to have been published by May 31, 2019. Application of the three strings yielded 33, 14, and 39 publications, respectively. We then performed a manual search of the references listed in publications found to discuss HSP in relation to the use of zebrafish. After excluding all articles not reporting direct research on zebrafish and HSP, 42 articles remained for inclusion in the review (see Figure 2 for a scheme of the methodology).

## Autosomal Dominant Complicated HSP Genes

To date, eight complicated HSP genes showing an autosomal dominant pattern of inheritance have been studied in zebrafish, namely: SPG3A, SPG4, SPG33, SPG10, SPG8, SPG17, SPG42, and SPG80.

### SPG3A

Fassier et al. (2010) generated an *atlastin-1* (*atl1*) morphant zebrafish model (mutations in *ATL1/*SPG3A are the second most common cause of HSP in humans). Atlastin-1 is a protein implicated in the vesicle trafficking and neurite outgrowth (Zhu, 2006; Orso et al., 2009; Kadnikova et al., 2019).

*ATL1* has been shown to be a major locus for early-onset autosomal dominant pure and complicated forms of HSP (Scarano et al., 2005; Fusco et al., 2010). More than 95% of individuals diagnosed with SPG3A have an affected parent, while the proportion of cases arising *de novo*, and is not fully known. The gene encodes a member of the dynamin superfamily of large GTPases (Zhao et al., 2001) and it is predominantly expressed in the mammalian brain, in particular at the level of hippocampus and pyramidal neurons (Zhu et al., 2003). Fassier et al. (2010) demonstrated expression of the gene at the level of motor neurons during early CNS development. Loss of *atl1* led to a dramatic reduction of larval motility in combination with increased branching of spinal motor axons (Table 1). This phenotype seemed to be specific to motor and cerebellar neurons and it was rescued by injection of human *ATL1* mRNA (Fassier et al., 2010). Therefore, overexpression of human *ATL1* or zebrafish *atl1* mRNA was shown to inhibit bone morphogenetic protein (BMP) signaling leading to complete loss of ventral structures. This idea was also supported by the co-localization of Alt1 and type I BMP receptor in endosomal structures along neurites. In addition, the phenotypes observed in *atl1* morphant embryos were recovered pharmacologically, by inhibiting the BMP pathway at the receptor level during the late embryonic stage. The recovery of the motility and axonal phenotype observed in this *atl1* knockdown model suggested that BMP plays a key role in *atl1-*dependent HSP (Fassier et al., 2010).

**Table 1:** Phenotype description of zebrafish models for HSP studies.

**Human genes**	**Zebrafish genes**	**Zebrafish modeling**	**Phenotype**			**References**
			**Life cycle**	**Behavior**	**Morphology**	
**AUTOSOMAL DOMINANT HSP GENES**						
SPG3A*/ATL1*	*atl1*	Morphant		Reduction of larval motility	Increased branching of spinal motor axons	Fassier et al., 2010
		Overexpression			Inhibition of BMP signaling with complete loss of ventral structures	Fassier et al., 2010
SPG4*/SPAST*	*spast*	Morphant		Impaired motility	Dramatic defects in motor axon outgrowth, curved tail, shorter and more disordered axons with aberrant position of branchiomotor neuron cell bodies	Wood, 2006
	*spast*	Morphant			Disorganized microtubule networks in the spinal cord, thinner microtubules in the spinal motor neuron axon	Julien et al., 2015
	*spast*	DrM1 morphant	Embryos failed to hatch from their chorion	Locomotion defects with reduced swimming speed and distances covered	Curved tail	Jardin et al., 2018
	*spast*	DrM61 morphant	Embryos failed to hatch from their chorion	Locomotion defects with reduced swimming speed and distances covered	Smaller eyes, yolk tube agenesis	Jardin et al., 2018
	*spast*	DrM1 and DrM61 mutant		Reduced swimming speed	Pathfinding defects of spinal motor neurons axons	Jardin et al., 2018
SPG33*/PROTRUDIN*	*protrudin*	Double morphant (*spast* and *protrudin*) together			Impaired head and yolk sac extension, curly tail	Zhang et al., 2012
SPG10*/KIF5A*	*kif5Aa*	Mutant	Larval lethality	Hyperexcitability, diminished touch response	Sensorimotor deficits, peripheral polyneuropathy, degeneration of peripheral sensory axons, inflated swim bladder, increased lateral, and dorsal pigmentation	Campbell et al., 2014
	*kif5Aa*	Mutant	Death at 10 days post-fertilization	Failed to inflate swim bladder	Darker embryos with expanded melanosomes	Auer et al., 2015
SPG8*/WASHC5*	*spg8*	Morphant			Slight or severe curly tail, enlarged heart cavities, abnormal development of motor neurons and interneurons in the spinal cord with irregular branching	Valdmanis et al., 2006
	*spg8*	Morphant		Short shiver response to tactile stimulation	Pericardial edema, severe skeletal muscle dysfunction, curved tails, reduction of the caudal neuronal tube, complete abolition of ventral and caudal motor neurons	Clemen et al., 2010
SPG17*/BSCL2*	*seipin*	Mutant		Decrease in spontaneous swimming	No motor neurons loss or morphological abnormalities	Holtta-Vuori et al., 2013
SPG42*/SLC33A1*	*slc33a1*	Morphant			Curly tails, defective axon outgrowth from spinal cord, scarce and poorly organized motor axons	Lin et al., 2008; Mao et al., 2015
SPG80*/UBAP1*	*ubap1*	Mutant			Misshapen axon and shorter motor neuron length	Farazi Fard et al., 2019
**AUTOSOMAL RECESSIVE HSP GENES**						
ALS2*/ALSIN*	*als2*	Morphant		Behavioral abnormalities, swimming defects	Irregular motor neuron outgrowth in the spinal cord	Gros-Louis et al., 2008
SPG11*/KIAA1840*	*spg11*	Morphant			Abnormal axon outgrowth, enlarged heart cavity, curly tail, deformities of the fin, CNS abnormalities	Martin et al., 2012
	*spg11*	Morphant			Perturbation of neuronal differentiation	Southgate et al., 2010
	*spg11*	Morphant		Loss of motility and paralysis	Accumulation of simple gangliosides in lysosomes	Boutry et al., 2018
*SPG15/ZFYVE26*	*suf*	Morphant		Motor impairment	Motor axon outgrowth failure	Martin et al., 2012; Kanagaraj et al., 2014
	*suf*	Mutant suf^(−/−)^	No phenotype	No phenotype	No phenotype	Kanagaraj et al., 2014
SPG39*/PNPLA6*	*pnpla6*	Morphant			Developmental abnormalities, fewer motor neurons, abnormal axons, curly tail, aberrant eyes, reduced optic vesicle size	Song et al., 2013
	*pnpla6*	Morphant			Body curvature, small head, and eyes	Hufnagel et al., 2015
SPG46*/GBA2*	*gba2*	Morphant		Locomotor phenotype	Curvy tail, axonal shortening/branching of motor neurons	Martin et al., 2013
SPG53*/VPS37A*	*vps37a*	Morphant		Striking and significant loss of motility		Zivony-Elboum et al., 2012
SPG76*/CAPN1*	*capn1a*				Abnormal branchiomotor neuron migration, disorganized axonal networks	Gan-Or et al., 2016
SPOAN*/KLC2*	*kcl2*	Overexpression morphant	High mortality		Curly-tail phenotype	Melo et al., 2015
*PCYT2*	*pcyt2*	Mutant *pcyt2_3*	Larval lethality			Vaz et al., 2019
	*pcyt2*	Mutant *pcyt2_13*			Smaller overall size and abnormal tail-fin morphology	Vaz et al., 2019
**OTHER FORMS OF HSP**						
*ARL6IP1, PGAP1*, and *USP8*	*arl6ip1, pgap1*, and *usp8*	Morphants		Reduction of larval movement	Curvy tail, abnormal branching of spinal motor neuron axons	Novarino et al., 2014
*MARS*	*mars*	Morphant			Too severe to be analyzed	Novarino et al., 2014
**X-LINKED FORMS**						
*SPG1/L1CAM*	*l1.1*	Morphant			Hydrocephalus, defects in axonal outgrowth, myelination abnormalities	Linneberg et al., 2019
SPG22*/MCT8*	*mct8*	Mutant *mct8^(−/−)^*		Impaired locomotor activity, decreased response to external stimuli	Defects in neural circuit assembly, alteration in the expression of myelin-related genes	Zada et al., 2014, 2016
**ATAXIA-SPASTIC SPECTRUM (ASS) GENES**						
SCA3*/ATXN3*	*sca3*	Mutant EGFP-Ataxin 3 84Q	Shorter mean survival time	Shorter swimming distance, signs of ataxin-3 neuropathology	Decreased axonal length of motor neurons	Watchon et al., 2017
	*sca3*	Mutant EGFP-Ataxin 3 23Q	Shorter mean survival time			Watchon et al., 2017
*ABCD1*	*abcd1*	Mutant abcd1sa509	Reduced larval survival	Decrease in larval evoked response, reduced swim distance, velocity, and time spent moving in spontaneous swimming in adult	Reduction of myelination of axons, increased apoptosis in the brain	Strachan et al., 2017
*EXOSC3*	*exosc3*	Morphant	Premature death	Poor motility	Small brain, short curved spine and reduced expression of dorsal hindbrain progenitor and cerebellar specific markers	Wan et al., 2012
*PRNP/Prp*	*prp1*	Morphant	High mortality		Defective midbrain and hindbrain development	Nourizadeh-Lillabadi et al., 2010
	*prp2*	Morphant	High mortality		Defective midbrain and hindbrain development, aberrant morphology of the trigeminal ganglion, reduced number of peripheral neurons	Nourizadeh-Lillabadi et al., 2010
*PSEN1* and *PSEN2*	*psen1, psen2*	Morphant			Shorter tail, disruption of posterior somite formation and smaller head-eye and total size, hydrocephalus	Campbell et al., 2006
	*psen1, psen2*	Double morphants			Embryos co-injected with Pen-2 MO and Pen-2 RNA appears morphologically normal	Campbell et al., 2006
	*psen1*	Morphant			Expanded hindbrain and midbrain ventricular spaces, absent yolk extension and increased yolk ball size, fewer and smaller melanocytes, decreased pigmentation, increased head angle, thinner spinal cord, smaller larva size, hydrocephalus	Nornes et al., 2008
	*psen2*	Morphant			Expanded hindbrain and midbrain ventricular spaces, absent yolk extension and increased yolk ball size, fewer and smaller melanocytes, decreased pigmentation, increased head angle, thinner spinal cord, smaller larva size, hydrocephalus	Nornes et al., 2008
	*psen1*	Morphant		Specific cognitive deficits in response behavior (reduced capacity to follow a non-aversive visual stimulus) and aversive behavior (reduced escape response)	Pericardial and brain edema, blood accumulation, aberrations in yolk extension, eye, and tail malformations	Nery et al., 2017
*NPC1*	*npc1*	Morphant	Premature death, before 2 dpf			Schwend et al., 2011
	*npc1*	Morphant	Premature death at 5 dpf			Louwette et al., 2013
	*npc1*	Mutant	High mortality during embryonic or juvenile stages	Ataxia symptoms in swimming at 4 dpf	Slower length growth, loss of Purkinje cells in cerebellum at 4 dpf	Lin et al., 2018
	*npc1*	Mutant	Early death between 8 and 12 dpf in larvae, inability to reproduce in adults	A swimming defect of balance with inability to maintain upright position in adult	Diffuse Purkinje cells, dark liver phenotype	Tseng et al., 2018

### SPG4 and SPG33

*SPAST* (the SPG4 gene) encodes spastin, a microtubule-severing protein that belongs to the AAA (ATPase associated with various cellular activities) family of ATPase and regulates the number and mobility of microtubules (Kadnikova et al., 2019). In addition, spastin is involved in the early secretory pathway and in BMP signaling (Evans et al., 2005; Salinas et al., 2005; Connell et al., 2009; Zhao and Hedera, 2013). Haploinsufficiency is the prevalent opinion as to the mechanism of the disease, but gain-of-function toxicity of the mutant proteins is another possibility (Qiang et al., 2019).

Mutations in SPG4 are associated with 40% of cases of autosomal dominant HSP. *SPAST* is required for motor axon morphogenesis and function during embryonic development. In zebrafish, *spast* morphants displayed dramatic defects in motor axon outgrowth and were morphologically abnormal with impaired motility (Table 1) compared with control embryos (Wood, 2006). Moreover, *SPAST* promotes neurite outgrowth in a protrudin (*ZFYVE27*)-dependent way. In fact, *ZFYVE27* (the SPG33 gene, also termed *PROTRUDIN*) is another gene whose mutations cause autosomal dominant HSP (Hashimoto et al., 2014). Double-morphant zebrafish (i.e., with mutation of both *spast* and *protrudin*) showed impaired head and yolk sac extension with a curly tail phenotype that was more severe than in fish injected with MOs specific for single transcripts. These phenotypes were partially rescued by wild-type human *SPAST* or *PROTRUDIN* overexpression (Zhang et al., 2012). Furthermore, a pharmacological study in *spast* morphants showed partial rescue of the morphological phenotype, the microtubule defects (Table 1), and the high levels of oxidative stress (typical of HSP disease) through ER-modulating drugs, such as methylene blue and salubrinal, and, to a lesser extent, guanabenz, and phenazine (Julien et al., 2015). In 2018, two *spast* isoforms (DrM1 and DrM61) were identified in zebrafish. Knockdown of each of these isoforms led to different motor neuron and locomotion defects, not rescued by the selective expression of the other isoform. Indeed, the expression and the distribution of the two isoforms were found to differ in zebrafish embryos. Isoform-specific knockdown led to morphants with distinctive characteristics: MO-*DrM1* morphants displayed a curved tail, whereas MO-*DrM61* morphants showed smaller eyes and yolk tube agenesis. In both cases, the embryos failed to hatch from their chorion and at t 72 hpf the larvae showed locomotion defects and secondary motor neuron axon pathfinding defects (Table 1). These results were more recently confirmed in *spast* mutant zebrafish strain harboring a truncating mutation after the second ATG codon, that impedes the synthesis of both DrM1 and DrM61 spastin isoforms (Table 1; Jardin et al., 2018).

### SPG10

SPG10*/KIF5A* is responsible for HSP in about 3% of families with a dominant form of the disease. *KIF5A* codes for the kinesin heavy chain of the main neuronal motor protein involved in long-distance axonal transport (Hirokawa et al., 2010). Due to a genome-wide duplication event, zebrafish possess two kinesin 5a genes, *kif5Aa* and *kif5Ab*, which have an overlapping expression that is strictly zygotic and neural specific (brain, spinal cord, and eye) (Campbell and Marlow, 2013). Mutations in *kif5Aa* (but not in *kif5Ab*) lead to larval lethality and sensorimotor deficits similar to those observed in human patients; moreover, zebrafish *kif5Aa* mutants were found to exhibit hyperexcitability and peripheral polyneuropathy as defective mitochondrial transport resulted in degeneration of peripheral sensory axons. At 6 dpf (days-post-fertilization), *kif5Aa* mutants had an inflated swim bladder, increased lateral and dorsal pigmentation, and a diminished touch response (Campbell et al., 2014). The other five kif5 zebrafish proteins were not able to rescue *kif5Aa* deficiency in mutants. Similarly, the generation of loss-of-function alleles of the anterograde motor protein *kif5Aa* resulted in darker embryos with expanded melanosomes within their melanocytes; the embryos failed to inflate the swim bladder and died at 10 dpf. Moreover mutant fish showed de-synchronization of retinal axon and tectal growth (Auer et al., 2015).

### SPG8

*WASHC5/*SPG8 encodes strumpellin, also known as SWIP. Strumpellin is implicated in endosomal trafficking and it is a direct interactor of valosin-containing protein (VCP). VCP is involved in protein aggregation in several neurodegenerative syndromes (Hirabayashi et al., 2001) and myofibrillar myopathies (Schröder and Schoser, 2009). In zebrafish, *spg8* morphant embryos show a slight or severe curly tail phenotype and enlarged heart cavities. Furthermore, knockdown embryos display abnormal development of motor neurons and interneurons in the spinal cord; both were found to be shorter with irregular branching (Valdmanis et al., 2006). Furthermore, *spg8* morphants developed pericardial edema, due to impaired ventricular contractility, and severe skeletal muscle dysfunction (Table 1), displaying a short shiver response to tactile stimulation (Clemen et al., 2010). Finally, morphant embryos showed a dramatic reduction of the caudal neuronal tube with complete abolition of the ventral and caudal motor neurons (Clemen et al., 2010).

### SPG17

*BSCL2* encodes seipin, an endoplasmic reticulum resident protein involved in lipid metabolism; mutations affecting its N-glycosylation lead to a dominantly inherited motor neuron disease, known as SPG17 or Silver syndrome (Warner et al., 2004). In this case, a zebrafish model has been used to test the effect of the “common” mutation p.N88S in seipin (Holtta-Vuori et al., 2013). Expression of the mutant protein in zebrafish led to a motility defect without motor neuron loss or morphological abnormalities. This reduction in swimming was paralleled by decreased triglyceride content in the developing head, possibly due to disturbances in the mobilization of yolk lipids. Oleic acid supplementation restored the motility defect of seipin-N88S-expressing fish. In this way, zebrafish have proven useful in showing that impairment of lipid metabolism is a contributing factor in the pathology associated with SEIPIN-N88S mutation (Holtta-Vuori et al., 2013).

### SPG42

*SLC33A1* encodes the endoplasmic reticulum (ER) membrane acetyl-CoA transporter, which is responsible for carrying acetyl-carboxylase (acetyl-CoA) into the ER lumen (Kanamori et al., 1997). Knockdown of the *slc33a1* gene in zebrafish using MOs generates embryos with curved tails and defective axon outgrowth from the spinal cord with scarce and poorly organized motor axons. This phenotype can be rescued by human wild-type mRNA, but not mutant mRNA (Lin et al., 2008). Moreover, Mao et al. (2015) discovered that *slc33a1* knockdown led to motor axonopathy through upregulation of BMP signaling, a pathway that regulates axonal growth, guidance and differentiation. Motor axon defects, consisting of shortened axons with increased axon branching, could be rescued by inhibition of BMP signaling by administration of dorsomorphin (DM), a drug that inhibits BMP receptor-1 activity (Mao et al., 2015). DM also promotes the differentiation of neuron progenitor cells from human pluripotent stem cells (PSCs) (Morizane et al., 2011) and the differentiation of cardiomyocytes from mouse and human PSCs (Hao et al., 2008; Kattman et al., 2011). Therefore, the zebrafish model of SPG42 has provided preliminary experimental support for the rationale of testing DM, with a view to proposing the drug for future trials in mammals.

### SPG80

Very recently, Farazi Fard et al. (2019) identified a novel autosomal-dominant gene responsible for HSP in 10 families of diverse geographic origin. SPG80 is a juvenile-onset neurological disorder characterized by a progressive spasticity and hyperreflexia. Some affected subjects also showed cerebellar signs and mild cognitive impairment (Farazi Fard et al., 2019). All affected individuals carried heterozygous non-sense or frameshift mutations in the *UBAP1* (ubiquitin-associated protein 1) gene. *UBAP1* is a member of the UBA domain family, which includes proteins that have a role in the ubiquitin and ubiquitination pathways (Qian et al., 2001).

Seeking to unveil the effects of mutations in *UBAP1 in vivo*, Farazi Fard et al. (2019) found that microinjection of CRISPR/Cas9 and sgRNAs against *UBAP1* in the transgenic fish with fluorescently labeled motor neuron Tg(Oligo2::DsRed) resulted in misshapen axons and shorter motor neuron length compared with what was observed in controls (Farazi Fard et al., 2019). This work further reinforced the suggestion that “UBAP1 links endosomal trafficking to the ubiquitination machinery pathways that have been previously implicated in HSPs.”

## Autosomal Recessive Complicated HSP Genes

Different genes have been associated with autosomal recessive (AR) HSP, all acting through loss-of-function and dominant-negative molecular mechanisms. Such mechanisms can be easily modeled at genetic level in zebrafish. There follows a brief overview of the AR-HSP genes studied in zebrafish.

### ALS2

Mutations in *ALS2*/alsin are associated with different neurodegenerative disorders, such as juvenile-onset amyotrophic or primary lateral sclerosis and AR hereditary spastic paraplegia. *ALS2* encodes for a protein (alsin) with multiple homology motifs similar to guanine-nucleotide exchange factor, indicating Rho and Rab5 activity. Members of the Rho and Rab5 family of GTPase proteins have been implicated in numerous cellular functions, the best characterized being regulation of the actin cytoskeleton (Rho) and protein trafficking through early endosomes (Rab5) (Otomo, 2003; Topp et al., 2004). *Als2* knockout mice developed rather normally and showed an only mild motor neuron phenotype (Cai, 2005; Yamanaka et al., 2006). Thus, in an attempt to mimic the disease phenotype, zebrafish *als2* morphant embryos were generated; these were found to show developmental and behavioral abnormalities (Table 1), which were rescued by overexpression of both full-length and novel *Als2* mouse transcripts (Gros-Louis et al., 2008). Hence, *alsin* might be implicated in motor axon pathfinding and thus be important in early development.

### SPG11

Hereditary spastic paraplegia with thin corpus callosum is a complicated form of AR-HSP linked to SPG11 mutations. The *KIAA1840*/SPG11 gene encodes spatacsin (Stevanin et al., 2007), whose mutations have been linked to spastic gait disorder, variably associated with cognitive impairment, peripheral neuropathy, cerebellar ataxia, parkinsonism, and retinal degeneration (Stevanin et al., 2007). Spatacsin is preferentially expressed in human and mouse cortical neurons and it is detected within neurites and growth cones and colocalizes with synaptic markers playing a role also in intracellular cargo trafficking (Pérez-Brangulí et al., 2014).

In mice, loss of *Spg11* led to early cognitive and motor deficits, consistent with the symptoms observed in the majority of patients with mutations in the SPG11 gene (Branchu et al., 2017). Loss of spatacsin impairs the formation of membrane tubules in lysosomes and cause lysosomal lipid accumulation altering the homeostatic equilibrium between cholesterol trafficking and cytosolic calcium levels (Boutry et al., 2019b). Downregulation of *spg11* in zebrafish resulted in abnormal axon outgrowth (Martin et al., 2012) and a range of developmental defects (Table 1) and CNS abnormalities. In general, morphant embryos exhibited perturbation of neuronal differentiation (Southgate et al., 2010). More recently, Boutry et al. (2018) demonstrated, both in mouse and in neuronal human cells, that loss of spatacsin led to accumulation of simple gangliosides in lysosomes due to impairment of their recycling. Altered lysosomal activity has also been observed in other models of HSP (Renvoisé et al., 2014; Allison et al., 2017), suggesting that the accumulation of undigested material could be responsible for a build-up of autophagy markers and thus for neurodegeneration. Moreover, Jeyakumar et al. (1999), using an antisense MO for *spg11*, showed that morphant larvae display a motor phenotype characterized by either a loss of motility or paralysis. This motor activity reduction was rescued by treating morphant larvae with miglustat, an FDA-approved drug that inhibits glucosyl-ceramide synthase, and is known to decrease GM2 ganglioside levels in a model of Sandhoff disease (Jeyakumar et al., 1999). This work offered new therapeutic strategies for neurodegenerative diseases linked to such lysosomal dysfunction (Boutry et al., 2018).

### SPG15

The *ZFYVE26*/SPG15 gene encodes spastizin; this locus was first reported to account for a rare form of spastic paraplegia, variably associated with mental retardation, hearing and visual defects, dysarthria, cerebellar signs, and distal amyotrophy, and sometimes referred to as Kjellin syndrome (Hanein et al., 2008; Goizet et al., 2009). Spastizin is a large protein (270 kDa) that localizes on the endoplasmic reticulum, on vesicles, and in the endosomal and lysosomal compartment. Spastizin plays a critical role in autophagy process and in particular in the formation and maturation of autophagosomes (Vantaggiato et al., 2019).

In cultured cells, spastizin co-localized partially with ER and endosome markers. This suggests that it may play a role in intracellular trafficking and that it is required for CNS function (Hanein et al., 2008). Clinically, SPG15 is very similar to SPG11 and recent studies have suggested interactions of the SPG15 and SPG11 proteins with the late endosomal/lysosomal adaptor protein complex AP-5 (Renvoisé et al., 2014). Functional studies in zebrafish showed that knockdown of the zebrafish SPG15 ortholog (*suf*) led to motor axon outgrowth failure and motor impairment (Martin et al., 2012). However, Kanagaraj et al. (2014) found that zygotic zebrafish *suf*^−/−^ embryos did not show a neurological phenotype or motor axon outgrowth failure, while they confirmed, after the MO injection, the previously reported severe phenotypes (Kanagaraj et al., 2014). These authors described an uncharacterized cellular mechanism for suf/spastizin activity during secretory vesicle maturation in zebrafish oogenesis, which raised the possibility of novel avenues for HSP therapy research (Kanagaraj et al., 2014).

### SPG39

Mutations in patatin-like phospholipase domain containing 6 (*PNPLA6*) have been implicated in a broad spectrum of neurodegenerative conditions including the complicated form of spastic paraplegia referred to as type 39 (SPG39). The SPG39 protein is a conservative protein found in many species ranging from yeast to mammals (Lush et al., 1998; Chang et al., 2008). Although its tissue distribution and function are well-established, its mechanism of action in the nervous system remains to be elucidated. Several animal models of *PNPLA6* mutation have been generated, including mice, fruit fly, and zebrafish. In particular, the zebrafish provided a vertebrate model able to better characterize the role of the protein in neural development. MO-induced loss of *pnpla6* in zebrafish caused developmental abnormalities (Table 1), which were rescued by overexpression of wild-type human *PNPLA6* mRNA (Song et al., 2013). Additionally, knockdown of *pnpla6* resulted in overactivation of BMP signaling, which can lead to impairment of axonal transport machinery and maintenance of long axons (Song et al., 2013). Hufnagel et al. (2015) identified eight mutations in the *PNPLA6* gene in six families with Oliver-McFarlane or Laurence-Moon syndrome and used zebrafish to functionally validate these mutations. They found that mutation-harboring mRNAs did not rescue the morphant phenotype, whereas wild-type human PNPLA6 mRNA did (Hufnagel et al., 2015). The discovery of these additional PNPLA6-opathies has considerable implications for the diagnostic and prognostic evaluation of patients with cerebellar or pyramidal dysfunction.

### SPG46

Autosomal recessive spastic paraplegia 46 (SPG46) is another neurodegenerative disorder characterized by onset in childhood of slowly progressive spastic paraplegia and cerebellar signs. Martin et al. (2013) identified, in three distinct families, four different mutations in *GBA2*, a gene that encodes for a microsomal non-lysosomal glucosylceramidase involved in lipid metabolism. Transient loss of the zebrafish *gba2* orthologous gene led to abnormal motor behavior and axonal shortening/branching of motor neurons. These phenotypes were rescued by co-injection of the human wild-type mRNA, but not by use of the same mRNA carrying the mutation. This study unveiled a new role of ceramide metabolism in HSP pathology (Martin et al., 2013).

### SPG53

As highlighted above, microtubule dynamics and vesicle trafficking play a critical role in HSP. Zivony-Elboum et al. (2012) demonstrated the involvement of *VPS37A* in an AR complicated HSP form (SPG53). *VPS37A* encodes a protein member of the endosomal sorting complex required for transport (ESCRT) system. The ESCRT system is involved in intracellular trafficking, in the maturation of multivesicular bodies, and in the sorting of ubiquitinated membrane proteins into internal luminal vesicles (Hurley, 2010). Zivony-Elboum et al. (2012) described 9 patients, from two different families, with early-onset spastic paraplegia in whom linkage analysis, followed by candidate gene sequencing, identified a homozygous mutation in *VPS37A*. Loss-of-function experiments using the MO strategy in zebrafish showed that morphant embryos presented striking and significant loss of motility compared with embryos injected with a standard control MO. Moreover, rescue experiments through co-injection of WT, but not mutated SPG53 mRNA, were able to restore the phenotype. These data suggested, and provided evidence for, the involvement of *VPS37A* in AR-HSP (Zivony-Elboum et al., 2012).

### SPG76

Another AR-HSP gene, this one responsible for spastic paraplegia 76 (SPG76), is *CAPN1*, identified by performing whole-exome sequencing in nine affected individuals from three families. *CAPN1* encodes calpain 1, a protease that is widely expressed in the CNS and has roles in synaptic plasticity, synaptic restructuring, and axon maturation and maintenance (Liu et al., 2008). Gan-Or et al. (2016) generated three models of calpain 1 deficiency, studying the effects of loss of function of *CAPN1* orthologs in nematode, fruit fly and zebrafish. In zebrafish, there are two orthologs of *CAPN1* (*cpn1a* and *capn1b*) but only the MO against *capn1a* led to a phenotype, because *capn1a* is largely expressed in the brain starting at 24 hpf (hours-post-fertilization) (Lepage and Bruce, 2008). Moreover, the authors injected the *capn1a*-MO in the Tg(islet1::GFP) zebrafish line expressing the green fluorescent protein (GFP) in the motor neurons, including the branchiomotor neurons. Capn1a deficiency resulted in abnormal branchiomotor neuron migration and disorganized axonal networks in the brain, supporting a neuroprotective role of calpain 1 (Gan-Or et al., 2016). This work opened up new avenues for expanding our knowledge about the role and effects of the different calpains on neurodegeneration and neuroprotection, and thus for furthering our understanding of the *CAPN1*-associated HSP phenotypes.

## SPOAN/*KLC2*

Twenty-six Caucasian individuals belonging to consanguineous families affected by a complicated form of AR spastic paraplegia, with optic atrophy and neuropathy (SPOAN syndrome) were identified in early 2000 (Macedo-Souza et al., 2009). Ten years later, Melo et al. (2015) described the SPOAN causative mutation, a small deletion in the non-coding region that causes kinesin light chain-2 (*KLC2*) overexpression. The zebrafish has proved to be an indispensable model for studying *in vivo* the effects of *klc2* knockdown and overexpression. Loss of function was obtained using two different *klc2* MOs (a translation-blocking MO and a splice MO); the effects of both MOs were evident at 48 hpf and the phenotypes observed were rescued by injection of WT *kcl2* mRNA. Moreover, the *kcl2* overexpression was associated with phenotypes similar to those displayed by morphants (Table 1) described in several reports that employed zebrafish to study other forms of HSP (Melo et al., 2015). The similar phenotypes, observed both in loss-of-function and in gain-of-function experiments, supported the idea that *kcl2* is an essential gene for motor neuron function and development (Melo et al., 2015).

### PCYT2

Very recently, Vaz et al. generated two distinct *pcyt2* knockout zebrafish lines, one targeting exon 3 *(pcyt2_03)* and the other targeting the final exon 13 *(pcyt2_13)* (Vaz et al., 2019). The gene *PCYT2* encodes phosphoethanolamine cytidylyltransferase, an enzyme in the phosphatidylethanolamine synthesis via, one of the most abundant membrane lipids presents in the brain (Nakashima et al., 1997). *Pcyt2*^−/−^ mice are embryonically lethal (Fullerton et al., 2007). In zebrafish, the survival analysis at both 5 days and 6 weeks, showed that the Complete abrogation of *pcyt2* (*pcyt2_03 line)* in zebrafish showed high mortality whereas *pcyt2_13* strain with some residual protein function revealed a higher survival compared to *pcyt2_03* line. Moreover, at 6 weeks of age, the *pcyt2_13* line showed smaller size and abnormal tail-fin morphology compared with the WT. This model highlighted that alterations in lipid metabolism may lead to complex HSP (Martin et al., 2013; Vaz et al., 2019).

## Other Forms of HSP

In 2014, Novarino et al. performed whole-exome sequencing in combination with network analysis in 55 families displaying AR-HSP and identified several previously unknown putative HSP genes. To establish the role of these disease genes in HSP, they studied their expression profiles, considering multiple human tissues, and then functionally validated many mutated genes by performing knockdown experiments in zebrafish, and generating fish models of *ARL6IPI, PGAP1, USP8*, and *MARS* deficiency by means of the MO strategy. Morphant larvae were evaluated for mortality, body axis defects, and altered motor neuron morphology, and were submitted to evoked and spontaneous swimming behavior analysis (Table 1; Novarino et al., 2014). Unfortunately, the *mars* morphants were too severe to be analyzed, whereas the phenotypes associated with the other genes were similar to those previously reported for other HSP candidate genes. The mutations and genes involved link HSP to cellular transport, nucleotide metabolism and synapse and axon development. Overall, this work led to the construction of a “HSPome” interaction map that may help guide future studies (Novarino et al., 2014).

### X-Linked Forms

Allan-Herndon-Dudley syndrome (AHDS), a rare disorder of brain development with neuromuscular involvement, is characterized by a combination of severe intellectual disability, spastic paraplegia, and disturbed thyroid hormone (TH) parameters (Bohan and Azizi, 2004). The AHDS locus was identified on chromosome X, and this condition occurs exclusively in young males. Bohan and Azizi (2004) suggested that AHDS was the fourth locus for X-linked spastic paraplegia, and proposed the term “SPG22” for the locus Xq21 that encompasses the AHDS region (Bohan and Azizi, 2004). AHDS is caused by mutations in *MCT8*, a gene that encodes a TH transporter. Since knockout mice were found to lack the neurological defects present in AHDS patients (Di Cosmo et al., 2010, 2013), Zada et al. (2016) created a *mct8* zebrafish mutant (*mct8*^−/−^) using a zinc-finger nuclease strategy. Zebrafish mutants showed neurological and behavioral alterations reminiscent of those seen in the human disease. Using several experimental approaches, the authors found abnormalities in the expression of myelin-related genes, defects in neural circuit assembly, a reduction of locomotor activity, and a decreased response to external stimuli. They suggested that these impaired neurological phenotypes and behaviors could be conserved between zebrafish and AHDS patients. In addition, the fact that treatment with TH analogs was able to recover a portion of the neurological phenotypes seems to pave the way for the use of zebrafish for large-scale testing of possible therapeutic treatments for AHDS pathology (Zada et al., 2014). Two years later, analysis of a mutant *mct8*^−/−^ zebrafish line focused on hypermyelination in the CNS of the mutant larvae, and showed a reduction in the number of oligodendrocytes in the brain and spinal cord, and an increase in the number of Schwann cells in the trunk (Zada et al., 2016). In addition, the authors evaluated the therapeutic effect of putative drugs, and it emerged that TH analogs and clemastine were able to partially restore hypomyelination in *mct8*^−/−^ mutants. Furthermore, the expression of mct8 was specifically targeted in the endothelial cells of the vascular system, with the outcome of a complete rescue of hypomyelinaton in *mct8*^−/−^ embryos before the maturation of the BBB. These data open the possibility of acting pharmacologically and genetically on hypomyelination in AHDS patients (Zada et al., 2016).

Jouet et al. showed that mutation in the *L1CAM* gene cause SPG1 and MASA syndrome characterized by corpus callosum aplasia, mental retardation, spasticity in the upper and lower limbs, and hydrocephalus (L1 syndrome) (Jouet et al., 1994; Fransen et al., 1995). Yu et al. revealed that *l1.1*, the ortholog of mammalian *L1CAM*, contributes to spinal cord regeneration in adult zebrafish (Chen et al., 2016). Recently, it has been shown that knockdown experiments of *l1.1* lead to hydrocephalus, defects in axonal outgrowth, and myelination abnormalities (Linneberg et al., 2019). These phenotypes have been previously associated with the L1 syndrome.

Another gene X-linked associated with spastic paraplegia is *PLP1* (SPG2) that encodes a primary constituent of myelin in the central nervous system. Few data are available on *plp1* in zebrafish (Brösamle, 2010) and no morphant investigations have been published.

## The Ataxia-Spasticity Spectrum Genes

The novel ASS classification proposed by Synofzik and Schüle (2017) considers 69 genes whose mutations are associated with both ataxia and spasticity. Among these genes, only 12 were previously classified as HSP (SPG) genes and 17 as SCA/SCAR genes (Synofzik and Schüle, 2017). Some SCA/SCAR genes included in the ASS classification, as well as other ASS genes not previously classified as HSP or HA genes have been modeled in zebrafish and hence they will here be described. Pathological polyQ expansion in the coding region of the *ATXN1* gene causes spinocerebellar ataxia type 1 (SCA1) (Klement et al., 1999). Two genes, designated *atxn1a* and *atxn1b*, were identified in zebrafish as homologs of human *ATXN1*. The products of *atxn1a* and *atxn1b* were expressed preferentially in the cerebellum, which is the main site of SCA1 disease (Carlson et al., 2009).

A similar study investigated the expression of *ataxin3*, the ortholog of *ATXN3* whose polyQ pathological expansion causes Machado-Joseph disease (MJD), also known as spinocerebellar ataxia type 3 (MJD/SCA3) (Matos et al., 2019). Recently, Watchon et al. created, as a model of SCA3 l, two transgenic zebrafish lines, expressing enhancer green fluorescence plasmid (EGFP). These lines were called EGFP-Ataxin 3-23Q and EGFP-Ataxin 3-84Q (Watchon et al., 2017). The two mutants showed a significantly shorter mean survival time, depending on the length of the polyQ expansion. The axonal length of motor neurons in 48 hpf larvae expressing EGFP-Ataxin 3-84Q was found to be decreased compared with what was observed in non-transgenic and EGFP-Ataxin 3-23Q-expressing larvae. The EGFP-Ataxin 3-84Q zebrafish also showed a significantly shorter swimming distance at 6 days and at 12 months of age compared with the other two groups, even when the human ataxin-3 protein was limited to motor neurons using a mir218-enhancer-Kal4 driver line (Watchon et al., 2017). The transgenic zebrafish also developed signs of ataxin-3 neuropathology. A neuritic beading-staining pattern (positive for ataxin-3 and polyQ), as previously observed in the medullary white matter of patients with MJD, was observed in the medulla of the EGFP-Ataxin 3-84Q zebrafish (Watchon et al., 2017). Interestingly, immunoblotting on protein lysate of transgenic zebrafish lines revealed the presence of ataxin 3-positive cleavage fragments as reported in MJD patient samples. Testing inhibitor compounds on these zebrafish lines, created as animal models of MJD, revealed that calpeptin, a calpain inhibitor, might represent a possible treatment, as it prevented the formation of the proteolytic cleavage products (Watchon et al., 2017).

Adrenoleukodystrophy (ADL) is another X-linked neurodegenerative disease. Caused by mutations in the *ABCD1* gene (Strachan et al., 2017), it affects the myelin of central and peripheral nervous systems. Mutations in *ABCD1* cause a spectrum of phenotypes ranging from cerebral forms of infantile adrenoleukodystrophy to adenomyeloneuropathy mimicking HSP. Zebrafish models of ADL were created by Strachan et al. (2017) through transcription activator-like effector nucleases in exon 1 of *abcd1* (*abcd1*^zc90^) and these authors also characterized a mutant model from the Zebrafish Mutation Project with a point mutation in *abcd1* exon 9 (*abcd1*^sa509^) (Strachan et al., 2017). The *abcd1*^sa509^ mutant showed increased levels of very long chain fatty acids in 7-8 dpf larvae, reduced myelinization of axons in 5 dpf larvae, and increased apoptosis in the brain at 72 hpf, not observed in *abcd1*^zc90^ mutants (Strachan et al., 2017). The *abcd1*^sa509^ 6 dpf mutants also showed reduced larval survival and a motor behavior deficit (Table 1) also seen in adults (Strachan et al., 2017).

In another study, Wan et al. (2012) used a zebrafish morphant to functionally examine the effects of mutations in exosome component 3 (*EXOSC3*), a gene associated with pontocerebellar hypoplasia 1 (PCH1) and distal hereditary motor neuronopathy, both of which resemble HSP. EXOSC3 is a component of the RNA exosome complex, and mutations in the relative gene were found in 13 families with signs of PCH1 (Wan et al., 2012). The *exosc3* knockdown model showed a severe phenotype at 3 dpf (Table 1; Wan et al., 2012). Other zebrafish gene orthologs in addition to those referred to in the group of ASS etiologies, including *prp1/2*, have also been studied and the main results are summarized in Table 1.

Among others, it is important to highlight *npc1*, the ortholog of the Niemann-Pick disease type C1 gene (*NPC1*). Mutations in this gene are responsible for 95% of cases of Niemann-Pick disease, a rare AR lysosomal storage disorder (Torres et al., 2017) with heterogeneous symptoms that include jaundice, hepatosplenomegaly, ataxia, spasticity, and intellectual decline leading to dementia (Schwend et al., 2011; Louwette et al., 2013; Lin et al., 2018; Tseng et al., 2018). The transmembrane protein NPC1 is implicated in the retrograde transport of cholesterol and glycolipids from lysosomes, and mutated NPC1 causes an accumulation of these substances in lysosomes, leading to onset of variable neurological manifestations (Lin et al., 2018; Tseng et al., 2018). The first studies of this gene in zebrafish were conducted using MOs, but morphants were characterized by premature death, before 2 dpf (Schwend et al., 2011) or at around 5 dpf when higher doses of MO were used (Louwette et al., 2013). The *npc1-*knockdown zebrafish showed developmental defects as reported in Table 1 (Schwend et al., 2011; Louwette et al., 2013). More recently, *npc1*^−/−^ zebrafish models were created using CRISPR/Cas9 technology (Lin et al., 2018; Tseng et al., 2018). Lin et al. observed that most *npc1*^−/−^ zebrafish with truncated Npc1 protein died during the embryonic or juvenile stages, and the few survivors showed slower length growth compared with the wild type; however, none survived after 8 dpf (Lin et al., 2018). At 4 dpf, survivors expressed ataxia symptoms in swimming that corresponded to a loss of Purkinje cells in the cerebellum (Lin et al., 2018). Concurrently, two other lines of *npc1* null mutants were created by Tseng et al. (2018). They observed similar results with massive early death between 8 and 12 dpf and a maximum lifetime of 6 dpf (Tseng et al., 2018). In this case, adult *npc1* mutants showed a swimming defect of balance with inability to maintain the upright position, likely due to a CNS defect more clearly observed in the Purkinje cells (Tseng et al., 2018). In addition, Tseng et al. observed a dark liver phenotype and inability to reproduce; they also found increased LysoTracker staining in neuromasts from 3 dpf null *npc1* mutants, a parameter that might be used as an *in vivo* screen for therapeutic drugs (Tseng et al., 2018).

## Conclusions and Future Applications

**Figure 3:**
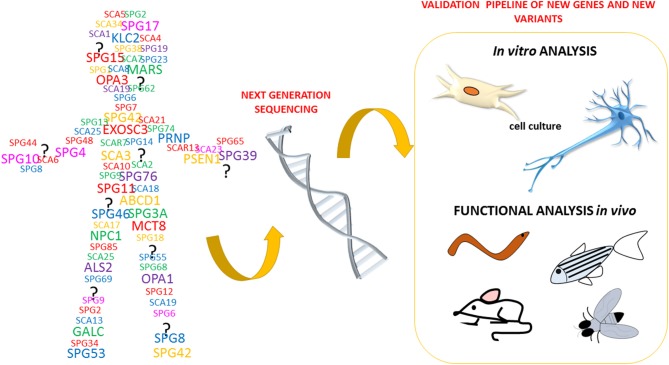
HSP phenotype identification and specificity assessment.

The recent technological advances in gene sequencing that allow the analysis of entire genomes of patients affected by neurodegenerative disorders, such as HSP and spastic ataxias (ASS) (Figure 3), have brought rapid developments in terms of the identification of novel genes and potentially pathogenic variants. This, in turn, has created a pressing need for functional characterization of these new variants and genes. To date, it clearly emerged that HSP, HA, ASS, and other neurological disorders are part of a continuum of overlapping clinical conditions. For instance, the clinical overlap of HSP with HA or intellectual/ developmental disability is not new and so it is the overlap with the mechanisms involved in more common neurological conditions including amyotrophic lateral sclerosis, multiple sclerosis, Parkinson disease, and dementias (Patten et al., 2014; Parodi et al., 2017; Boutry et al., 2019b; Shribman et al., 2019). Since HSP and ASS are a group of disorders characterized by high genetic diversity, researchers have widely exploited the zebrafish as an *in vivo* model system of these pathologies.

In this review, we have highlighted some of the key features that make the zebrafish a compelling organism for modeling complicated forms of HSP and ASS. Zebrafish has been used to study molecular and functional aspects of HSP and ASS *in vivo*, as well as to functionally validate new mutations identified in patients. Even though the main publications in this field focus on loss-of function experiments performed using the MO strategy, research has now started to exploit the possibility of creating stable mutant lines to be used to better identify the mechanisms of complicated forms of HSP, and as a tool for drug screening. Taken together, these approaches have resulted in the development of gene-specific of HSP and ASS zebrafish models.

**Figure 4:**
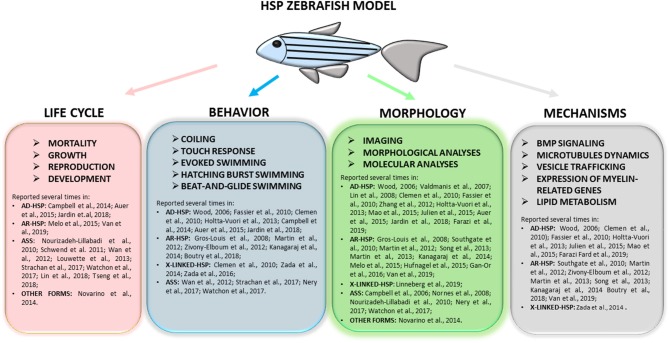
Full overview on the wide range of tests applied to research complicated HSP phenotypes in zebrafish and of molecular mechanisms recurrent in different models. Refer to the literature mentioned in the text for specific protocols.

This literature review highlights several common phenotypes among the different disease models thus far described: almost all showed locomotor impairment, motor neuron defects, and body malformations. However, these phenotypes can also be relevant to other forms of neurodegeneration impacting on upper and lower motor neurons and long neuronal tracts. As outlined in Figure 3, our review shows that certain zebrafish phenotypes are common traits in the complicated forms of HSP but also that there is not yet a straightforward readout correlating functional studies with mutations in HSP and ASS genes (Table 1). Obviously, zebrafish cannot be expected to fully replicate complex human disorders. Thus far, efforts to identify HSP traits in zebrafish models have involved the use a wide range of tests (behavioral, life cycle, and morphological) (Figure 4), but there is a great variability between studies, due to the intrinsic limits of the tests chosen, and also because, in many cases, the analyses, being based on operator choice, are biased. Standardization of the processes used to characterize zebrafish phenotypes would certainly help to better define ones corresponding to neurodegenerative diseases in humans, including HSP and related conditions, and would clearly improve the validity of this model system and likely facilitate its use in drug screening.

## Author Contributions

FS, VN, and MM: conceptualization. VN and MM: methodology. GF, SM, and VN: investigation. GF and VN: resources and writing original draft preparation. FS, MM, AO, AD, FG, and VN: writing review and editing. SM: visualization. FS and MM: supervision and funding acquisition. VN: project administration.

### Conflict of Interest

The authors declare that the research was conducted in the absence of any commercial or financial relationships that could be construed as a potential conflict of interest.
